# Adolescent Solastalgia and Climate Distress in Colombia: Psychosocial Correlates Across Urban, Semiurban, and Rural Contexts

**DOI:** 10.3390/ijerph23070884

**Published:** 2026-07-09

**Authors:** Marcela Guapacha-Montoya, Felipe Agudelo-Hernández

**Affiliations:** 1 Pediatrics Department, Facultad de Ciencias Para la Salud, Universidad de Caldas, Manizales 170004, Colombia; 2 Facultad de Ciencias para La Salud, Universidad de Manizales, Manizales 170001, Colombia; afagudeloh81703@umanizales.edu.co

**Keywords:** adolescent, climate change, mental health, environmental exposure

## Abstract

**Highlights:**

**Public health relevance—How does this work relate to a public health issue?**

- Environmental degradation and climate-related territorial change are emerging determinants of adolescent mental health, particularly in settings marked by social inequality and limited access to care.
- This study examines solastalgia as a place-based form of psychological distress among school-attending adolescents living in urban, semiurban, and rural Colombian territories affected by environmental transformation.

**Public health significance—Why is this work of significance to public health?**

- The findings show that adolescent solastalgia is associated with psychosocial, health-related, school-related, and territorial factors, rather than being explained only by individual psychological vulnerability.
- By focusing on adolescents in the Global South, the study contributes evidence from underrepresented populations where climate change, environmental injustice, extractivism, and mental health risks often overlap.

**Public health implications—What are the key implications or messages for practitioners, policy makers and/or researchers in public health?**

- School-based mental health strategies in environmentally affected territories should include climate- and environment-sensitive screening, culturally safe listening spaces, and referral pathways for adolescents experiencing distress.
- Public health responses should connect adolescent mental health care with environmental protection, water security, territorial justice, and intersectoral action involving schools, health services, communities, and environmental authorities.

**Abstract:**

**Background**: Environmental degradation and climate-related territorial transformation are increasingly recognized as social determinants of adolescent mental health. However, empirical evidence on solastalgia among young people in the Global South remains limited. This study aimed to examine psychosocial, health-related, school-related, family, and territorial factors associated with solastalgia among school-attending adolescents in Colombia. **Methods**: A quantitative, cross-sectional analytical study was conducted between August 2025 and February 2026 with 1005 adolescents from three Colombian territories undergoing different forms of environmental transformation: an urban setting affected by construction-related change, a semiurban setting affected by agricultural transformation, and a rural setting affected by extractivism. Solastalgia was assessed using the Brief Solastalgia Scale. Psychosocial, health-related, school-related, and contextual variables were measured using validated or locally supported instruments. Bivariate analyses, multiple linear regression, and non-parametric territorial comparisons were conducted. **Results**: Solastalgia differed significantly across territorial groups, with the semiurban group showing the highest mean ranks for solastalgia, followed by the rural and urban groups. However, territorial comparisons showed heterogeneous psychosocial and health-related patterns rather than a uniform gradient across all outcomes. Older age was independently associated with higher solastalgia scores. In the final regression model, solastalgia was independently associated with resilience, substance use screening, suicide risk, age, physical health status, conflict resolution, concern about climate change, and territorial origin. The model explained 42.9% of the variance in solastalgia scores. Territorial origin contributed a small but statistically significant additional proportion of explained variance. **Conclusions**: Adolescent solastalgia in Colombia is not reducible to individual psychological vulnerability. It reflects a broader socioecological configuration involving emotional distress, perceived health, psychosocial resources, school-related experiences, concern about climate change, and place-based environmental transformation. These findings support the need for school-based and community-based public health responses that integrate adolescent mental health care with environmental protection, cultural safety, and territorial justice.

## 1. Introduction

Adolescent mental health is a global public health priority. Adolescence is a formative period in which emotional regulation, social relationships, educational trajectories, and coping strategies are consolidated; therefore, family, school, community, and territorial conditions play a central role in present and future well-being. Globally, one in seven adolescents aged 10–19 years experiences a mental disorder, and depression, anxiety, and behavioural disorders are among the leading causes of illness and disability in this population [1].

In this context, the climate crisis and environmental degradation are increasingly recognized as determinants of mental health. Climate change can affect psychological well-being through direct exposure to extreme events, heat, pollution, displacement, food insecurity, loss of livelihoods, community disruption, and anticipatory concern about the future. These effects are unevenly distributed: children, adolescents, Indigenous Peoples, rural communities, impoverished populations, and historically excluded territories often face higher exposure and weaker institutional protection [2].

Recent evidence shows measurable associations between environmental exposures and mental health outcomes in young people. A systematic review and meta-analysis found that high temperatures were associated with increased risk of mental health-related hospital visits or hospitalizations, depression, composite mental health outcomes, and suicide risk among children, adolescents, and young adults. These findings suggest that the environment is not merely a background condition, but a material determinant that can shape sleep, concentration, daily routines, physical activity, use of public space, mood, and everyday opportunities for well-being [3].

Climate anxiety has also become an important concept for understanding young people’s emotional responses to the climate crisis. It refers to persistent worry, fear, helplessness, sadness, or functional impairment associated with the perception of current or future climate threats. Climate anxiety should not automatically be pathologized; it can be a proportionate response to a real threat and may mobilize care, agency, and collective action. However, it may become harmful when combined with hopelessness, low perceived agency, lack of social support, institutional inaction, or the sense that the future has been compromised by adult, political, or economic decisions over which young people have little control [4,5,6].

Several concepts have been proposed to describe emotional responses to environmental degradation. Although related, they are not interchangeable. Climate anxiety refers mainly to fear, worry, or distress regarding climate change and its consequences. Ecological grief describes grief in response to experienced or anticipated ecological losses, including the loss of species, ecosystems, meaningful landscapes, and ways of life connected to place. Solastalgia, in contrast, refers to distress experienced when people remain in their home environment, but that environment changes in ways that undermine familiarity, safety, continuity, or meaning [7,8].

Solastalgia is particularly useful for understanding loss without physical displacement. Unlike nostalgia, which usually refers to longing for a place from which one is absent, solastalgia occurs when home changes while one continues to inhabit it. For adolescents, this is especially relevant because territory shapes peer relationships, family memories, school routines, community trajectories, future expectations, and identity. River degradation, forest loss, pollution, agricultural transformation, urban expansion, and extractivism may therefore be experienced not only as environmental damage, but also as disruptions to everyday life and to the possibility of imagining a future in one’s own place.

This requires moving beyond the idea of nature as an external “context” separated from social life. Humans, institutions, infrastructures, animals, rivers, soils, crops, climate, and technologies form assemblages that shape concrete ways of inhabiting, caring, suffering, and becoming ill. In this sense, the ecological crisis is not only an environmental crisis, but a reorganization of the political and material conditions of existence; human and environmental systems must be understood as historically entangled and mutually constitutive [9,10].

This approach also connects with One Health and planetary health perspectives, which understand human health as inseparable from animal, ecosystem, climate, and social health. Environmental crises are not external threats to human well-being, but transformations of the interdependent systems that sustain life: water, food, biodiversity, soils, animals, institutions, local economies, and community ties. The One Health Commission has emphasized systems thinking, equity, epistemic pluralism, and socioecological sustainability; similarly, planetary health scholarship has called for integrated approaches to climate change, biodiversity loss, and the social determinants of health [11,12].

Adolescent solastalgia can be understood as a situated expression of a broader socioecological crisis. It is not merely an individual reaction to environmental deterioration, but a form of distress produced at the intersection of climate crisis, food insecurity, contamination, territorial inequality, institutional erosion, and loss of the material conditions needed to sustain everyday life. This reading is close to the concept of polycrisis, in which multiple crises become causally entangled and amplify one another’s effects, and to global climate-health frameworks that call for health-centred, equity-oriented responses to irreversible environmental harms [13,14].

This perspective avoids reducing environmental distress to an individual psychological response. It frames distress as a socially situated experience produced by inequalities, territorial histories, development models, institutional decisions, and unequal forms of exposure. From an ecosocial epidemiological perspective, environmental harm can be understood as embodied history: bodies express, through health and distress patterns, the material and symbolic conditions in which people live [15,16].

In Indigenous, rural, and Global South contexts, solastalgia requires conceptual care. A decolonial and Indigenist perspective emphasizes that territory is not only a physical space or economic resource, but a living relation that connects memory, spirituality, food, kinship, authority, mobility, care practices, and cultural continuity. Environmental loss can therefore involve ecological harm, psychosocial suffering, food insecurity, community disruption, and threats to cultural reproduction at the same time [17].

### 1.1. Socioecological Mechanisms Affecting Youth Mental Health

Solastalgia, environmental distress, and climate anxiety may affect adolescent mental health through multiple, interdependent, and situated processes. These should not be understood as linear causal mechanisms demonstrated by the present study, but as plausible theoretical pathways that guide variable selection, interpretation of associations, and contextual reading of findings. This distinction is important because, in a cross-sectional design, it is methodologically appropriate to speak of associated configurations rather than causality.

Environmental degradation changes the material conditions of daily life. Temperature, air quality, water availability, food security, contaminant exposure, mobility, access to green spaces, outdoor time, and the possibility of sustaining community or recreational practices may all be altered by environmental transformation. Among adolescents, these conditions may affect sleep, concentration, mood, physical activity, perceived safety, school performance, and emotional regulation [3].

Environmental distress also emerges because environmental change interferes with the ordinary reproduction of life. It can affect how young people get to school, what they eat, what water they drink, which places feel safe, which activities disappear, what conversations circulate within families, and what futures seem possible. In communities closely dependent on territory, ecological change can simultaneously affect household economies, intergenerational knowledge, food security, local identity, care practices, and mental health [18].

Solastalgia becomes especially relevant when a place is no longer recognizable, reliable, or emotionally habitable. For adolescents, territory is not only physical space: it organizes belonging, family memory, peer relations, school routines, mobility, and future expectations. When landscapes change due to mining, pollution, urbanization, deforestation, or agricultural transformation, the continuity between past, present, and future is disrupted. This loss may be expressed as sadness, anger, fear, helplessness, uprooting, or the sense that local futures are narrowing [19].

These experiences are not merely individual. Environmental losses can disrupt practices that sustain identity and belonging, such as farming, fishing, recognizing rainfall cycles, using medicinal plants, bathing in rivers, moving through the territory, participating in rituals, or listening to elders’ stories. In Indigenous and rural communities, these practices are not cultural accessories but conditions of collective continuity. Youth mental health must therefore be understood in relation to the possibility of sustaining ties with territories, memories, practices, and relationships that give meaning to community life [17,20].

Solastalgia and climate anxiety may intensify when adolescents perceive environmental deterioration not as accidental, but as the result of political, economic, or corporate decisions that unevenly affect certain territories. In such cases, suffering does not arise only from ecological loss but from feelings of abandonment, injustice, and lack of protection. When a territory is perceived as disposable, environmental experience becomes a moral and political experience as well [21].

Evidence on youth climate conversation spaces suggests that listening, emotional validation, and community building can support well-being without requiring every emotion to be immediately converted into activism [22]. In the present study, this literature was used to frame solastalgia as a socioecological, relational, and territorially situated phenomenon. The analytical question was therefore not only which adolescents reported higher solastalgia, but under which configurations of territory, environmental exposure, social support, school, family, belonging, and institutional trust, environmental deterioration was associated with higher distress.

### 1.2. Environmental Injustice, the Global South, and Polycrisis

The climate and environmental crisis unfold within a global structure of inequality. Populations that have contributed least to environmental degradation often face the highest exposure and the weakest institutional response. In the Global South, environmental impacts combine with poverty, informality, barriers to healthcare, educational inequality, environmental racism, armed conflict, food insecurity, institutional fragility, and dependence on extractive economies. These are not separate crises; they reinforce one another.

The concept of polycrisis helps explain how multiple systems in crisis become entangled and generate effects that are more severe than the sum of their parts. In this sense, adolescent solastalgia in Colombia can be read as a situated expression of coupled crises: environmental crisis, food crisis, mental health crisis, institutional trust crisis, territorial crisis, and care crisis. This approach avoids reducing the problem to a simple relationship between “damaged environment” and “symptoms” and instead highlights how suffering emerges within interdependent social and ecological systems [13].

The right to water is central to this discussion. Environmental public health recognizes water, sanitation, air quality, chemical safety, and climate action as necessary conditions for well-being. In the Americas, environmental risks account for a substantial burden of disease and death; moreover, substances such as pesticides, lead, and mercury disproportionately affect children and pregnant women [23]. Chocó clearly illustrates the convergence between environmental injustice, territory, and mental health. Mercury mining, river contamination, food insecurity, displacement, armed violence, and the weakening of cultural practices are not independent exposures, but intertwined dimensions of vulnerability. In Colombian Indigenous communities, solastalgia has been associated with food insecurity and other mental health problems; recent research in Indigenous paediatric populations has also linked the climate crisis, food security, and emotional problems in highly vulnerable territorial contexts [24].

This reading also applies to semiurban and urban territories. In Caldas, construction projects, urban expansion, and agricultural transformation may modify landscapes, water sources, local livelihoods, productive dynamics, and perceived environmental safety. In urban contexts, environmental distress may not appear as forest or river loss, but as cumulative transformation of the built environment: heat, noise, dust, reduced green areas, pollution, real estate pressure, or loss of meaningful places. Evidence from Medellín shows that environmental effects are lived through rhythms, bodies, and situated urban experiences, beyond conventional exposure indicators [25].

### 1.3. The Present Study 

A developmental perspective is also needed because adolescence is not a universal experience detached from place, history, culture, and structural inequality. Research on adolescent development has increasingly argued that the field remains shaped by Euro-American epistemologies and by evidence produced mainly in Minority World settings, even though most adolescents live in Africa, Asia, the Middle East, Latin America, and the Caribbean. For studies on climate-related distress, this imbalance is especially problematic: adolescents in the Majority World often experience environmental transformation alongside extractivism, poverty, food and water insecurity, violence, and limited access to mental health care. Therefore, examining solastalgia among Colombian adolescents contributes not only to climate mental health research but also to a more contextually grounded and decolonial developmental science in which territory, culture, and environmental injustice are treated as central to adolescent well-being [26].

Despite growing research on climate anxiety, ecological grief, and solastalgia, important gaps remain. Much of the evidence comes from high-income countries, adult populations, or studies focused on extreme climate events. More research is needed with adolescents in the Global South, rural territories, Indigenous contexts, populations affected by extractivism, and school settings. It is also necessary to understand how psychosocial factors may buffer or amplify environmental distress.

In Colombia, this gap is especially relevant. The country combines high ecological and cultural diversity with territorial inequality, extractive economies, socioenvironmental conflicts, agricultural transformation, urban expansion, barriers to mental healthcare, and differential exposure to violence. Studying solastalgia among Colombian adolescents allows the field to move toward a situated, intercultural, and socioecological perspective. It also makes it possible to examine whether environmental distress differs according to territory, relationship with place, and the family and school conditions available to cope with environmental change.

Based on the above, the objective of this study was to analyse psychosocial factors associated with levels of solastalgia among school-attending adolescents from three Colombian populations with different territorial contexts-urban, semiurban, and rural-and different forms of environmental transformation, to identify differential patterns of environmental distress according to territory of residence. The main hypothesis was that levels of solastalgia among Colombian adolescents would be associated with psychosocial factors, and that these associations would differ according to territorial context. Specifically, lower perceived social support, lower sense of territorial belonging, greater emotional distress, and concern about climate change were expected to be associated with higher levels of solastalgia. It was also hypothesized that adolescents from rural and semiurban contexts might present differential patterns of solastalgia compared with adolescents from urban contexts, due to different forms of relationship with territory, exposure to environmental transformation, and school conditions specific to each setting.

## 2. Materials and Methods

### 2.1. Study Design

A quantitative, cross-sectional, analytical study was conducted between August 2025 and February 2026 to analyse psychosocial and school-related factors associated with solastalgia among school-attending adolescents from three Colombian populations: one urban, one semiurban, and one rural. The cross-sectional design allowed solastalgia levels, psychosocial characteristics, and participants’ sociodemographic characteristics to be measured at a single point in time. Given the nature of the design, the results were interpreted as statistical associations rather than causal relationships.

### 2.2. Territorial Context

The study was conducted in three main educational institutions located in territories undergoing differentiated processes of environmental transformation. The first institution was in an urban population in the department of Caldas, exposed to environmental modifications associated with construction projects. The second institution was in a semiurban population in northern Caldas, where territorial changes are related to agricultural productive transformations. Caldas has a diversified agricultural structure that includes coffee, plantain, panela cane, avocado, citrus fruits, cacao, and other crops. In addition, Hass Avocado has been prioritized in municipalities such as Aranzazu and Anserma, with productive expansion projects and environmental concerns related to water use, erosion, impacts on water sources, and changes in areas traditionally recognized for their coffee-growing vocation or role in water protection.

The third institution was in a rural population in the department of Chocó, a territory with strong ecological, cultural, and community dependence on the river, the forest, and traditional livelihoods. In Indigenous communities in Chocó, especially among the Emberá Dobidá, the literature has described impacts derived from mercury mining in the Atrato River, environmental contamination, spiritual and physical rupture with the territory, food insecurity, forced displacement, and exposure to violence, creating a context in which environmental degradation, extractivism, and mental health intersect [27].

The inclusion of these three contexts allowed for comparison among adolescents living in territories with different relationships with the environment, different school conditions, and diverse forms of exposure to environmental transformation. This comparison was central to evaluating whether solastalgia and its associated factors vary according to the territorial context of residence.

### 2.3. Participants and Sample

The study population consisted of all adolescents enrolled in the three participating schools. A census sampling strategy by institution was used, inviting all eligible students from each school to participate. Adolescents were included if they had informed consent from their caregivers and provided their own informed assent. Participation was voluntary, confidential, and had no academic, disciplinary, or institutional consequences. In each institution, less than 2% of invited students declined participation or did not complete the consent and assent requirements. Records without consent or assent, questionnaires with insufficient information to calculate the main solastalgia variable, and cases with critical inconsistencies in identification, institution, or territorial classification variables were excluded. Territorial categories were not treated as ethnic categories; they referred to school and socioenvironmental settings. Therefore, urban, semiurban, and rural groups should not be interpreted as mutually exclusive cultural or ethnic populations. Individual-level ethnic self-identification was not available in the cleaned analytic dataset and was not used in the statistical models.

### 2.4. Instruments 

The dependent variable was the solastalgia score, understood as the emotional distress associated with the perceived deterioration, loss, or negative transformation of the inhabited environment. The independent variables included psychosocial, school-related, and family factors: depressive symptoms, anxiety, positive and negative suicidal ideation, resilience, life satisfaction, emotional regulation, frustration tolerance, conflict resolution, psychoactive substance use, and concern about climate change. Sociodemographic and contextual covariates were also included, such as age, sex, school grade, family composition, educational institution, perceived health status, access to health services, and type of territory: urban, semiurban, or rural.

Solastalgia was assessed using the Brief Solastalgia Scale -BSS-, a brief instrument designed to measure the negative emotional experience associated with environmental transformation. The scale was adapted and validated in Colombian Indigenous communities with histories of extractivism, including Wayuú populations affected by coal mining and Emberá Dobidá populations in Chocó affected by mercury mining. The Colombian study reported content validity through Aiken’s V values ranging from 0.80 to 0.99, an adequate unidimensional structure, and empirical reliability of 0.857 [28]. Concern about climate change was measured with a single binary item referring to recent emotional experience. Adolescents were asked whether, during the past four weeks, they had felt worried about climate change or about its effects on the place where they live. Responses were recorded dichotomously as yes/no and coded as 1 for the presence of climate change concern and 0 for its absence.

Depressive symptoms were assessed using the Patient Health Questionnaire-9 (PHQ-9), a nine-item scale that measures depressive symptoms over the previous two weeks, including anhedonia, depressed mood, sleep and appetite disturbances, fatigue, feelings of worthlessness, difficulty concentrating, psychomotor changes, and thoughts of death or self-harm. In Colombia, adequate validity has been reported for depression screening in primary care, with Cronbach’s alpha = 0.80, McDonald’s omega = 0.81, and an area under the curve of 0.92 [29].

Positive and negative suicidal ideation was assessed using the Positive and Negative Suicide Ideation Inventory -PANSI-, a 14-item scale that simultaneously assesses negative suicidal thoughts and protective factors or reasons for living. Its use is relevant because it does not reduce suicide risk to the presence of negative ideation alone but also allows for the identification of protective cognitive resources [30].

Resilience was assessed using the Connor-Davidson Resilience Scale -CD-RISC-10-, which measures perceived capacity for coping, adaptation, and recovery in the face of adversity [31]. For Colombia, the available psychometric reference corresponds to adults [32]. Since that validation was not conducted specifically with adolescents, internal reliability was estimated in the present adolescent sample, yielding high internal consistency (Cronbach’s α = 0.92). Therefore, the CD-RISC-10 was considered reliable for the analyses in this study, although it is acknowledged that the previous Colombian validation was conducted in an adult population.

Psychoactive substance use was assessed using the CRAFFT scale, a brief screening instrument designed for adolescents. The scale identifies patterns of alcohol and other substance use through questions on use in risky contexts, personal consequences, and signs of problematic use. In Colombia, the scale was validated in adolescents aged 14 to 18 years by comparison with a clinical interview as the reference criterion; the study reported sensitivity of 0.95, specificity of 0.83, positive predictive value of 0.85, and negative predictive value of 0.94 for a cut-off point ≥ 2 [33].

Life satisfaction was assessed using the Satisfaction with Life Scale -SWLS-, a five-item scale that measures the global cognitive evaluation people make of their own lives [34]. Colombian evidence has reported good psychometric properties for the SWLS, including a unidimensional structure, factorial equivalence with Spanish samples, and adequate internal consistency [35]. Frustration tolerance and conflict resolution were assessed using school-based social competence scales derived from the Colombian model Competencia Social y Salud Escolar (School Social Competence and Health) [36]. Frustration tolerance assesses recognition of frustration, self-control, rule-following, and search for alternatives; in this study, Cronbach’s alpha was 0.87, and the factor analysis supported a single dimension. Conflict resolution assesses conflict identification, negotiation, empathy, and coping; in this study, Cronbach’s alpha was 0.79, and the factor analysis also supported a single dimension.

All scores were interpreted according to the direction of each measure. Higher BSS, PHQ-9, and PANSI scores indicated higher solastalgia, depressive symptoms, and suicide risk, respectively. Higher SWLS, CD-RISC-10, frustration tolerance, and conflict resolution scores indicated more favourable psychosocial resources. Perceived physical and mental health were coded so that higher values reflected better perceived health. The CRAFFT variable was retained as a screening score based on the coding available in the dataset; lower values reflected greater endorsement of substance-use risk responses, whereas higher values reflected fewer endorsed risk responses. This coding direction was considered when interpreting correlations, regression coefficients, and mean-rank comparisons.

### 2.5. Procedure

Data collection took place between August 2025 and February 2026, in coordination with the participating educational institutions and the health secretariats of each region. Before administering the instruments, the study objective was presented to school administrators, teachers, caregivers, and students. It was explained that participation was voluntary, that responses would be confidential, and that students could withdraw at any time without academic or institutional consequences.

Caregivers signed informed consent, and adolescents provided informed assent. The questionnaires were administered in school spaces designated to ensure privacy, comprehension of the questions, and support from the research team when needed. Teachers, administrators, and other school authority figures were not allowed to access students’ individual responses. The three regions received support for an intercultural school mental health model, in coordination with the territorial health secretariats. This support was described as an ethical, community-based mechanism for activating care pathways, not as an evaluated intervention. Its purpose was to ensure that the identification of emotional symptoms, suicidal ideation, substance use, or other psychosocial needs could be promptly referred to school psychosocial teams and territorial health care pathways.

### 2.6. Data Organization and Analysis

Data cleaning included review of duplicates, verification of valid ranges, identification of impossible values, review of inconsistencies between age and school grade, removal of records without consent or assent, calculation of missing data by participant and by scale, and review of potentially invalid response patterns. For missing data, the proportion of missingness by variable and scale was examined. When the proportion of missing data was low, complete-case analysis was used. All statistical analyses were performed using R software, version 4.6.1 (R Core Team, R Foundation for Statistical Computing, c/o Institute for Statistics and Mathematics, Wirtschaftsuniversität Wien, Welthandelsplatz 1, 1020 Vienna, Austria).

Normality of continuous variables was assessed through graphical inspection and appropriate statistical tests. Internal reliability of the applied scales was assessed using Cronbach’s alpha. To evaluate factors associated with solastalgia, regression models were constructed with the solastalgia score as the dependent variable. The main model included psychosocial factors and sociodemographic covariates. Model assumptions were assessed through residual analysis, evaluation of multicollinearity using variance inflation factors, identification of influential observations, and review of the linearity of associations.

Comparisons were then conducted among the three territorial groups: urban, semiurban, and rural. For continuous variables, the Kruskal–Wallis test was used, and post hoc pairwise comparisons were conducted using the Mann–Whitney U test, with correction for multiple comparisons when necessary. The effect size for non-parametric between-group comparisons was calculated from the Z statistic of the Mann–Whitney U test, using the formula r = Z/√N, where N corresponds to the total number of observations included in each comparison. This estimate allowed statistical significance to be complemented with a measure of the magnitude of the observed differences.

### 2.7. Ethical Considerations

The study was conducted in accordance with ethical principles for research involving human participants of the Declaration of Helsinki, with particular attention to the protection of children and adolescents. The protocol received institutional ethics approval [Anonymized]. Participation was voluntary and was preceded by informed consent from caregivers and informed assent from adolescents. Participants were informed that they could withdraw from the study at any time, without academic, disciplinary, institutional, or service-access consequences. Information was handled confidentially, and identification codes were used to protect students’ identities. The study incorporated criteria for cultural safety, recognizing that the relationship with territory, the experience of environmental transformation, and the ways distress is expressed may vary among adolescents from urban, semiurban, rural, and intercultural contexts. Therefore, the instruments were administered using understandable language, with support when needed, and with respect for community and territorial particularities.

## 3. Results

### 3.1. Sample Characteristics

The final analytic sample included 1005 school-attending adolescents from urban, semiurban, and rural territories in Colombia. Participants had a mean age of 14.51 years (SD = 3.49). Most adolescents came from the urban setting, followed by the rural and semiurban settings. The sample included Indigenous and Afro-descendant adolescents, as well as participants who reported no ethnic affiliation. Gender distribution was relatively balanced, with a slight predominance of male participants and a small proportion of non-binary or gender-diverse adolescents. Territorial categories were analysed as socioenvironmental contexts, not as ethnic groupings. Sociodemographic data are presented in Table 1.

When participants were grouped into early adolescence (11–13 years; n = 344), middle adolescence (14–16 years; n = 586), and late adolescence (17–19 years; n = 75), mean solastalgia scores increased from 11.48 (SD = 5.32) to 12.79 (SD = 4.91) and 13.45 (SD = 4.55), respectively. Older adolescents also showed higher resilience scores, whereas depressive symptoms were highest in the 14–16-year group.

Bivariate analyses showed that solastalgia was significantly associated with selected psychosocial, health-related, and contextual variables. The strongest positive association was observed with resilience, followed by territorial origin, suicide risk, conflict resolution, and depressive symptoms. Solastalgia was also negatively associated with the CRAFFT screening score, physical health status, and mental health status. Because higher CRAFFT values reflected fewer endorsed substance-use risk responses, this negative association indicated that higher solastalgia was related to greater substance-use screening risk, poorer perceived physical health, and poorer perceived mental health.

No significant associations were observed between solastalgia and several school-related variables, including friends at school and bullying. Likewise, life satisfaction and frustration tolerance were not significantly correlated with solastalgia at the bivariate level. Overall, the correlation pattern suggests that solastalgia was more consistently related to emotional, health-related, and territorial variables than to most school-functioning indicators. Full bivariate correlations are presented in Table 2.

### 3.2. Factors Associated with Solastalgia

A multiple linear regression model was estimated to identify factors independently associated with solastalgia. The final model was statistically significant, F(8, 992) = 93.16, *p* < 0.001, and explained 42.9% of the variance in solastalgia scores (R^2^ = 0.429, adjusted R^2^ = 0.424). The standard error of the estimate was 7.18. The Durbin–Watson statistic was 1.84, suggesting no relevant evidence of autocorrelation in the residuals.

As shown in Table 3, solastalgia was associated with a combination of psychosocial, health-related, school-related, and territorial variables. The final model retained resilience, CRAFFT screening score, suicide risk, age, physical health status, conflict resolution, concern about climate change, and territorial origin as independent predictors. The inclusion of territorial origin contributed a small but statistically significant additional proportion of explained variance. If ΔR^2^ is retained as 0.004, the corresponding value would be approximately F change (1, 992) = 6.95, *p* = 0.009. This supports the relevance of territorial context in explaining differences in solastalgia. Overall, the model suggests that solastalgia among adolescents was not explained by a single individual-level factor, but by a broader configuration of emotional, health-related, school-related, and territorial variables. However, although the model explained a substantial proportion of variance, other contextual, environmental, cultural, and community-level factors not included in the model may also contribute to adolescent solastalgia.

### 3.3. Territorial Comparisons

Pairwise Mann–Whitney U tests showed significant territorial differences across several health, psychosocial, substance-use screening, and solastalgia-related variables. Mean ranks were interpreted according to the coding direction of each measure; therefore, higher ranks did not have the same substantive meaning across all outcomes. The semiurban group showed the highest solastalgia mean ranks, followed by the rural and urban groups. However, the semiurban group showed the lowest mean ranks for perceived physical health, perceived mental health, frustration tolerance, conflict resolution, and the CRAFFT screening score. Because lower CRAFFT values indicated greater endorsement of substance-use risk responses, this pattern suggests higher substance-use screening risk in the semiurban group. Depression mean ranks were highest in the rural group, whereas suicide-risk mean ranks were higher in the urban group than in the semiurban and rural groups. Thus, the territorial pattern was not a uniform hierarchy of better or worse functioning; instead, each setting showed a different configuration of distress, resources, perceived health, and environmental concern (Table 4).

## 4. Discussion

This study examined psychosocial and territorial factors associated with solastalgia among adolescents living in urban, semiurban, and rural Colombian contexts exposed to different forms of environmental transformation. Overall, the findings partially supported the initial hypothesis. Solastalgia differed significantly across territorial groups, with the semiurban group showing the highest mean ranks, followed by the rural and urban groups. In the regression model, territorial origin remained independently associated with solastalgia, although its additional contribution to explained variance was small. The model explained 42.9% of the variance in solastalgia, suggesting that environmental distress was shaped by a configuration of psychosocial, health-related, and territorial variables rather than by a single domain.

Consistent with the hypothesis, solastalgia was associated with emotional distress indicators, including depressive symptoms and suicide risk. Some associations required careful interpretation of scale direction. Resilience, conflict resolution, and concern about climate change were positively associated with solastalgia, suggesting that environmental distress may coexist with awareness, coping resources, and social engagement. The negative association with the CRAFFT screening score should not be read as lower substance-use risk; given the coding direction, it indicates that adolescents with higher solastalgia tended to endorse more substance-use risk responses. These findings support an interpretation of solastalgia as a complex socioecological experience rather than a simple marker of individual psychological vulnerability.

The territorial pattern is one of the central findings of the study. The higher solastalgia mean ranks observed in the semiurban group may reflect the instability of transitional territories, where agricultural transformation, changing land use, and perceived environmental uncertainty may be highly visible in everyday life. The rural group also showed higher solastalgia than the urban group, which is consistent with the strong dependence of rural communities on land, water, forest, and traditional livelihoods. However, the fact that the semiurban group showed the highest levels suggests that solastalgia may not only emerge from severe environmental degradation, but also from accelerated or ambiguous territorial change, especially when communities perceive that familiar landscapes, livelihoods, or future possibilities are being reconfigured. 

The positive association between resilience and solastalgia is important and should be interpreted cautiously. Rather than indicating that resilience increases distress, it may suggest that adolescents with greater coping resources, environmental awareness, or connection to place are also more capable of recognizing and reporting environmental loss. Similarly, the positive association with conflict resolution may indicate that more socially engaged adolescents are more exposed to collective narratives about territorial change. The CRAFFT finding also becomes more coherent once coding direction is considered: lower screening scores reflected greater endorsement of substance-use risk responses, and these lower scores were associated with higher solastalgia. This pattern should still be explored through sensitivity analyses and qualitative inquiry, but it no longer needs to be interpreted as a contradiction in the main findings.

These findings are consistent with a growing body of international literature showing that climate change and environmental degradation affect young people’s mental health through both direct exposure and indirect socioecological pathways [21]. Evidence from systematic and meta-analytic studies has shown associations between heat exposure and mental health outcomes, including depression, hospital visits, and suicide risk among children and adolescents [3]. Research on youth climate emotions has also emphasized that distress is not limited to anxiety, but includes sadness, anger, helplessness, grief, and future-oriented uncertainty [5,37].

From a decolonial developmental perspective, this supports the need to avoid applying generic models of adolescent distress derived from Minority World contexts to Global South settings. Instead, adolescent solastalgia should be read as a situated developmental experience shaped by place, intergenerational memory, school life, environmental risk, age-related expectations, and unequal territorial futures [26]. The findings also align with studies showing that solastalgia can mediate the relationship between environmental disruption and mental health outcomes, and that both lived and anticipated environmental change may produce distress, anger, grief, and a sense of betrayal when valued environments are transformed [38,39,40].

The results also resonate with systems-oriented approaches to climate-related youth mental health, which argue that young people’s distress cannot be adequately understood at the individual level alone, but must be situated within family, school, institutional, ecological, and political systems [21,41]. From a place-health perspective, the findings also support the idea that health and well-being are shaped by attachment to place, loss of meaningful landscapes, environmental dispossession, and culturally specific relationships with territory [42,43].

In Colombia and Latin America, these findings are particularly relevant because environmental transformation is often embedded in broader histories of inequality, extractivism, violence, food insecurity, and limited access to mental health care. The results are consistent with recent Colombian studies showing that solastalgia is a useful construct for understanding the suffering of Indigenous children and young people exposed to extractivism, displacement, food insecurity, and weakening of cultural practices [27]. They also align with evidence from Colombian Indigenous communities linking solastalgia with mental health problems and food insecurity, especially in territories affected by mining and environmental degradation [24]. The Chocó context is especially important because mercury contamination, illegal mining, river degradation, and the disruption of river-based livelihoods make environmental distress inseparable from water justice, cultural survival, and collective health.

At the regional level, studies in Latin America have shown that climate change, health, migration, and territorial vulnerability are deeply interconnected, and that climate-related risks are amplified by poverty, institutional weakness, and social exclusion [44]. The Colombian urban comparison also matters. Environmental suffering is not limited to rural or Indigenous territories, since urban exposures such as air pollution, heat, noise, reduced green space, and infrastructural transformation may also be embodied in everyday rhythms and health experiences [25].

The environmental justice implications of these results are not limited to distributive injustice, understood as unequal exposure to environmental harm. They also involve procedural and recognitional injustice: adolescents experience changes in rivers, crops, neighborhoods, school surroundings, and community futures, but they often have limited power to participate in decisions that define land use, water protection, mining control, urban expansion, or local environmental recovery. This is especially relevant for interpreting the link between solastalgia and disrupted continuity between past, present, and future. Askland’s concept of eritalgia is useful here because it extends the nostalgia-solastalgia dyad toward the suffering produced when place-based futures are compromised by externally imposed or extractive transformations [45]. In this study, the semiurban pattern and the age gradient suggest that distress may emerge not only from present degradation but also from the perception that familiar and desired futures in place are becoming less attainable.

### 4.1. Implications

The findings have implications for school mental health, environmental health policy, territorial planning, and environmental education. Solastalgia should be recognized as a legitimate form of adolescent distress related to environmental transformation, not merely as an individual emotional problem. School-based mental health systems in environmentally affected territories should include climate- and environment-sensitive screening, culturally safe listening spaces, teacher training, and referral pathways for adolescents with depressive symptoms, suicidal ideation, substance-use risk, or severe distress. Classrooms can also function as spaces where environmental mental health is discussed without pathologizing youth emotions, while connecting emotional expression with environmental knowledge, collective care, and local action.

Responses should not focus only on individual coping. They should also strengthen community participation, environmental protection, water security, and intersectoral action between schools, health secretariats, environmental authorities, and local communities. From an environmental justice perspective, this means creating mechanisms through which adolescents can participate in discussions about ecological risks, school environments, water protection, territorial planning, and community futures. This is consistent with recommendations to integrate mental health into climate change responses, strengthen community-based mental health systems, reduce vulnerability, and support climate-resilient health services [2]. A rights-based approach is also needed, particularly in territories affected by water contamination, mercury exposure, extractivism, or environmental injustice, where the protection of health requires action on the environmental determinants of health, not only access to clinical care [23].

### 4.2. Limitations

Several limitations should be considered. The cross-sectional design does not allow causal inference or temporal ordering between environmental distress and psychosocial variables. All measures were self-reported, which may introduce recall bias, social desirability bias, or differential reporting across territories and cultural groups. Although the sample used a census strategy within the participating schools, it does not represent adolescents who were out of school or absent during data collection. 

The territorial variable captured broad urban, semiurban, and rural contexts, but did not include objective environmental exposure measures such as water quality, air pollution, land-use change, proximity to mining, or satellite-based indicators of environmental degradation. The unexpected negative association between substance use screening and solastalgia also requires caution and should be explored through sensitivity analyses, review of coding direction, and possibly qualitative inquiry. Finally, multiple pairwise comparisons increase the risk of type I error, although effect sizes were reported to complement statistical significance.

## 5. Conclusions

This study contributes evidence on solastalgia among adolescents in Colombia by showing that environmental distress varies across urban, semiurban, and rural territories and is associated with a broader configuration of psychosocial, health-related, and territorial factors. The findings support a socioecological interpretation of solastalgia: it is not only an individual emotional response, but a situated experience shaped by environmental transformation, place-based relationships, perceived health, school life, and social vulnerability. The territorial differences observed suggest that adolescent solastalgia may be especially relevant in contexts where environmental change is visible, ongoing, and connected to uncertainty about the future. Addressing this form of distress requires school-based, community-based, culturally safe, and rights-oriented responses that connect mental health care with environmental protection and territorial justice.

## Figures and Tables

**Table 1:** Descriptive and Sociodemographic Data.

Variable	Mean	SD
PHQ-9—Depression	9.34	7.35
Frustration Tolerance (FT)	10.02	2.42
Conflict Resolution (CR)	9.97	2.42
Resilience	27.43	11.34
Solastalgia (BSS)	12.82	7.91
Substance Use Screening (CRAFFT)	16.40	6.59
Satisfaction With Life Scale (SWLS)	23.75	7.89
Suicide Risk (PANSI)	31.67	8.71
	Frequency	%
Origin		
Urban	617	61.39
Semi-urban	166	16.52
Rural	222	22.09
Ethnicity and territory		
Indigenous—urban	35	3.48
Indigenous—semiurban	60	5.97
Indigenous—rural	73	7.26
Afro-descendant—urban	20	1.99
Afro-descendant—semiurban	62	6.17
Afro-descendant—rural	168	16.72
No ethnic affiliation	587	58.41
Gender		
Male (Cisgender/Transgender)	527	52.44
Female (Cisgender/Transgender)	468	46.57
Non-binary/Gender Diverse	10	1.00
Education level		
Fifth	6	0.60
Sixth	176	17.51
Seventh	136	13.53
Eighth	160	15.92
Ninth	252	25.07
Tenth	124	12.34
Eleventh	134	13.33
Twelfth	17	1.69
Who do you live with?		
Parents	334	33.23
Mother	165	16.42
Grandparents	255	25.37
Father	37	3.68
Other relatives	90	8.96
I live alone	5	0.50
I prefer not to answer	106	10.55
Other	1	0.10
Single father	12	1.19
Religion		
None	29	2.89
Catholic	639	63.58
Christian	337	33.53
Physical health		
Very poor	51	5.07
Poor	103	10.25
Fair	168	16.72
Good	520	51.74
Very good	163	16.22
Mental health		
Very poor	49	4.88
Poor	145	14.43
Fair	192	19.10
Good	481	47.86
Very good	138	13.73
Concern about climate change		
Yes	501	49.85
No	398	39.60
I prefer not to answer	106	10.55
Bullying		
Yes	280	27.86
No	675	67.16
I prefer not to answer	50	4.98
Difficulties with math processes		
Yes	296	29.45
No	680	67.66
I prefer not to answer	29	2.89
Difficulties understanding instructions		
Yes	291	28.96
No	693	68.96
I prefer not to answer	21	2.09
Confusing letters when writing or reading		
Yes	230	22.89
No	764	76.02
I prefer not to answer	11	1.09

**Table 2:** Correlations Between Variables.

Variable	Physical Health	Mental Health	Concern About Climate Change	Friends at School	Bullying	Extracurricular Activities	Confusion with Instructions	Confuses Letters When Reading	Difficulties in Mathematics	Depression	Frustration Tolerance	Conflict Resolution	Life Satisfaction	Suicide Risk	CRAFFT Screening Score	Resilience	Solastalgia
CC Origin	−0.339 **	−0.256 **	−0.067 *	0.034	−0.136 **	0.065 *	−0.044	−0.017	−0.037	0.218 **	−0.143 **	−0.161 **	−0.012	−0.170 **	−0.528 **	0.070 *	0.134 **
Sig.	<0.001	<0.001	0.035	0.286	<0.001	0.041	0.168	0.58	0.244	<0.001	<0.001	<0.001	0.695	<0.001	<0.001	0.026	<0.001
CC Physical Health		0.607 **	−0.087 **	−0.042	0.131 **	−0.171 **	0.024	0.037	0.073 *	−0.059	0.174 **	0.129 **	0.065 *	0.120 **	0.363 **	−0.189 **	−0.125 **
Sig.		<0.001	0.006	0.181	<0.001	<0.001	0.438	0.245	0.02	0.061	<0.001	<0.001	0.038	<0.001	<0.001	<0.001	<0.001
CC Mental Health			−0.120 **	−0.065 *	0.138 **	−0.083 **	0.068 *	0.074 *	0.103 **	−0.146 **	0.121 **	0.197 **	0.194 **	0.135 **	0.327 **	−0.108 **	−0.090 **
Sig.			<0.001	0.039	<0.001	0.009	0.032	0.02	0.001	<0.001	<0.001	<0.001	<0.001	<0.001	<0.001	0.001	0.004
CC Climate change				0.078 *	0.016	0.103 **	−0.112 **	−0.042	−0.095 **	0.168 **	0.054	−0.012	−0.195 **	−0.126 **	−0.001	−0.071 *	0.022
Sig.				0.013	0.607	0.001	<0.001	0.188	0.003	<0.001	0.086	0.709	<0.001	<0.001	0.967	0.025	0.48
CC Friends					0.042	0.086 **	−0.002	0.018	−0.023	0.055	−0.073 *	−0.099 **	−0.094 **	−0.085 **	−0.015	−0.024	−0.02
Sig.					0.179	0.006	0.96	0.573	0.466	0.082	0.021	0.002	0.003	0.007	0.642	0.448	0.522
CC Bullying						0.03	0.032	0.092 **	0.105 **	−0.203 **	−0.013	0.01	0.102 **	−0.024	0.075 *	0.043	−0.054
Sig.						0.337	0.313	0.004	0.001	<0.001	0.676	0.743	0.001	0.444	0.017	0.171	0.085
CC Extra Activities							−0.028	−0.04	−0.084 **	0.063 *	−0.019	0.035	−0.047	−0.076 *	−0.110 **	0.036	0.073 *
Sig.							0.379	0.201	0.008	0.046	0.543	0.268	0.133	0.016	<0.001	0.256	0.02
CC Instructions								0.246 **	0.279 **	−0.234 **	−0.075 *	−0.002	0.195 **	0.019	0.036	0.107 **	0.004
Sig.								<0.001	<0.001	<0.001	0.017	0.937	<0.001	0.538	0.257	0.001	0.904
CC Letter Confusion									0.116 **	−0.172 **	−0.097 **	−0.011	0.112 **	−0.107 **	0.036	0.116 **	−0.017
Sig.									<0.001	<0.001	0.002	0.724	<0.001	0.001	0.249	<0.001	0.588
CC Math										−0.198 **	−0.023	−0.03	0.127 **	−0.029	0.098 **	0.109 **	<0.001
Sig.										<0.001	0.464	0.337	<0.001	0.351	0.002	0.001	0.988
CC Depression											0.164 **	0.021	−0.348 **	0.06	−0.123 **	−0.281 **	0.106 **
Sig.											<0.001	0.511	<0.001	0.055	<0.001	<0.001	0.001
CC Tolerance												0.294 **	0.014	0.138 **	0.158 **	−0.105 **	0.039
Sig.												<0.001	0.669	<0.001	<0.001	0.001	0.213
CC Resolution													0.087 **	0.206 **	0.137 **	−0.032	0.108 **
Sig.													0.006	<0.001	<0.001	0.316	0.001
CC Satisfaction														0.109 **	0.038	0.261 **	0.059
Sig.														0.001	0.228	<0.001	0.061
CC Suicide															0.128 **	0.151 **	0.116 **
Sig.															<0.001	<0.001	<0.001
CC CRAFFT score																−0.175 **	−0.173 **
Sig.																<0.001	<0.001
CC Resilience																	0.225 **
Sig.																	<0.001

Note. * The correlation is significant at the 0.05 level. ** The correlation is significant at the 0.01 level (two-tailed). CC = correlation coefficient.

**Table 3:** Regression Model for Solastalgia.

Variable	B	SE B	Beta	t	*p*	Lower Limit (95%)	Upper Limit (95%)	Tolerance	VIF
(Constant)	1.653	1.993		0.829	0.407	−2.258	5.564		
Resilience	0.16	0.021	0.23	7.633	<0.001	0.119	0.202	0.906	1.104
CRAFFT screening score	−0.16	0.04	−0.133	−4.036	<0.001	−0.238	−0.082	0.755	1.325
Suicide Risk	0.129	0.027	0.142	4.697	<0.001	0.075	0.183	0.897	1.115
Age	0.159	0.035	0.13	4.507	<0.001	0.09	0.228	0.983	1.018
Physical Health Status	−0.661	0.238	−0.088	−2.777	0.006	−1.128	−0.194	0.824	1.214
Conflict Resolution	0.277	0.097	0.085	2.846	0.005	0.086	0.468	0.923	1.083
Concern climate change	−0.952	0.343	−0.081	−2.778	0.006	−1.624	−0.279	0.972	1.029
Place of Origin	0.7	0.312	0.073	2.248	0.025	0.089	1.312	0.781	1.281

Note. Unstandardized coefficients are reported as B, with standard errors shown as SE B. Standardized coefficients are shown as β. The model was statistically significant, F(8, 992) = 93.16, *p* < 0.001, R^2^ = 0.429, adjusted R^2^ = 0.424. Collinearity diagnostics are reported using tolerance and VIF.

**Table 4:** Pairwise territorial comparisons of health, psychosocial, and solastalgia outcomes.

Variable	Comparison	Mean Rank Group 1	Mean Rank Group 2	U	Z	*p*	r
Physical health status	Urban vs. Semiurban	467.43	111.64	4670.5	−19.443	<0.001	0.61
	Urban vs. Rural	441.46	360.37	55,249.0	−4.916	<0.001	0.15
	Semiurban vs. Rural	101.02	264.40	2908.0	−14.672	<0.001	0.46
Mental health status	Urban vs. Semiurban	460.62	133.07	8228.5	−17.661	<0.001	0.56
	Urban vs. Rural	429.38	390.24	61,880.5	−2.302	0.021	0.07
	Semiurban vs. Rural	102.87	263.01	3216.0	−14.379	<0.001	0.45
Depression	Urban vs. Semiurban	404.67	344.92	43,395.5	−3.029	0.002	0.10
	Urban vs. Rural	372.75	551.32	39,333.5	−9.430	<0.001	0.30
	Semiurban vs. Rural	133.64	240.01	8323.5	−9.253	<0.001	0.29
Frustration tolerance	Urban vs. Semiurban	422.90	277.14	32,144.0	−7.461	<0.001	0.23
	Urban vs. Rural	430.88	389.75	61,771.0	−2.199	0.028	0.07
	Semiurban vs. Rural	163.56	217.64	13,290.0	−4.758	<0.001	0.15
Conflict resolution	Urban vs. Semiurban	424.62	270.77	31,087.0	−7.867	<0.001	0.25
	Urban vs. Rural	433.32	382.99	60,271.0	−2.686	0.007	0.09
	Semiurban vs. Rural	162.38	218.52	13,094.5	−4.952	<0.001	0.15
Life satisfaction	Urban vs. Semiurban	391.52	393.78	50,915.5	−0.114	0.909	0.01
	Urban vs. Rural	422.53	412.97	66,926.0	−0.505	0.614	0.01
	Semiurban vs. Rural	197.95	191.92	17,853.5	−0.524	0.600	0.01
Suicide risk	Urban vs. Semiurban	411.65	318.98	39,089.0	−4.693	<0.001	0.14
	Urban vs. Rural	441.71	359.67	55,094.5	−4.332	<0.001	0.14
	Semiurban vs. Rural	189.58	198.18	17,609.5	−0.748	0.454	0.01
CRAFFT screening score	Urban vs. Semiurban	462.68	129.30	7603.5	−17.066	<0.001	0.54
	Urban vs. Rural	482.37	246.66	30,006.5	−12.683	<0.001	0.40
	Semiurban vs. Rural	152.25	226.09	11,412.5	−6.470	<0.001	0.21
Resilience	Urban vs. Semiurban	335.94	600.39	16,619.0	−13.382	<0.001	0.42
	Urban vs. Rural	435.84	375.97	58,712.0	−3.159	0.002	0.10
	Semiurban vs. Rural	277.04	132.78	4724.5	−12.542	<0.001	0.40
Solastalgia	Urban vs. Semiurban	365.27	491.36	34,717.5	−6.402	<0.001	0.20
	Urban vs. Rural	408.29	452.54	61,263.5	−2.341	0.019	0.07
	Semiurban vs. Rural	221.69	174.17	13,912.0	−4.139	<0.001	0.13

Note. MR = mean rank. Pairwise comparisons were conducted using Mann–Whitney U tests. Higher ranks should be interpreted according to the direction of each scale. Higher perceived physical and mental health, resilience, frustration tolerance, conflict resolution, and life satisfaction scores indicate a more favourable status. Higher depression, suicide-risk, and solastalgia scores indicate more symptoms, risk, or distress. For the CRAFFT screening score, lower values reflected greater endorsement of substance-use risk responses; therefore, lower mean ranks indicate greater substance-use screening risk.

## Data Availability

The raw data supporting the conclusions of this article will be made available by the authors on request.
